# Metabolic Changes in Masseter Muscle of Rats Submitted to Acute Stress Associated with Exodontia

**DOI:** 10.1371/journal.pone.0128397

**Published:** 2015-06-08

**Authors:** Mamie Mizusaki Iyomasa, Fernanda Silva Fernandes, Daniela Mizusaki Iyomasa, Yamba Carla Lara Pereira, Rodrigo Alberto Restrepo Fernández, Ricardo Alexandre Calzzani, Glauce Crivelaro Nascimento, Christie Ramos Andrade Leite-Panissi, João Paulo Mardegan Issa

**Affiliations:** 1 Department of Morphology, Physiology and Basic Pathology, Ribeirão Preto Dentistry Faculty, University of São Paulo, Ribeirão Preto, 14040–904, SP, Brazil; 2 Biology Dental Buco Graduate Program, School of Dentistry of Piracicaba, University of Campinas, Piracicaba, 13414–903, SP, Brazil; 3 Department of Physiology, Medical School, University of São Paulo, Ribeirão Preto, 14040–900, SP, Brazil; 4 Psychobiology Graduate Program, School of Philosophy, Science and Literature of Ribeirão Preto, University of São Paulo, Ribeirão Preto, 14040–901, SP, Brazil; University of Nebraska-Lincoln, UNITED STATES

## Abstract

Clinical evidence has shown that stress may be associated with alterations in masticatory muscle functions. Morphological changes in masticatory muscles induced by occlusal alterations and associated with emotional stress are still lacking in the literature. The objective of this study was to evaluate the influence of acute stress on metabolic activity and oxidative stress of masseter muscles of rats subjected to occlusal modification through morphological and histochemical analyses. In this study, adult Wistar rats were divided into 4 groups: a group with extraction and acute stress (E+A); group with extraction and without stress (E+C); group without extraction and with acute stress (NO+A); and control group without both extraction and stress (NO+C). Masseter muscles were analyzed by Succinate Dehydrogenase (SDH), Nicotinamide Adenine Dinucleotide Diaphorase (NADH) and Reactive Oxygen Species (ROS) techniques. Statistical analyses and two-way ANOVA were applied, followed by Tukey-Kramer tests. In the SDH test, the E+C, E+A and NO+A groups showed a decrease in high desidrogenase activities fibers (P < 0.05), compared to the NO+C group. In the NADH test, there was no difference among the different groups. In the ROS test, in contrast, E+A, E+C and NO+A groups showed a decrease in ROS expression, compared to NO+C groups (P < 0.05). Modified dental occlusion and acute stress - which are important and prevalent problems that affect the general population - are important etiologic factors in metabolic plasticity and ROS levels of masseter muscles.

## Introduction

Orofacial pain is a condition associated with hard and soft tissues of the head, face, neck and all intra-oral structures [1]. Among different types of musculoskeletal and craniofacial pain, temporomandibular disorder (TMD) is prevalent in dental clinics and involves clinical problems in masticatory muscles and temporomandibular joints (TMJ) [2, 3]. In addition, it is known that TMD can be related to pain in other parts of the body [4]. It is important to note that TMJ pain can result in important psychological impacts [5]. Indeed, nociceptive and emotional behaviors are related and are widely represented by overlapping neural pathways, which may form substrates for pain modulatory effects of emotions [6–8].

Patients with TMD reveal high levels of stress, anxiety and depression[9]. Some studies have shown emotional states altering oral functions in animals and humans [10]. There are indications that acute stress can induce an increase in biting behaviors in rats [11]. Furthermore, routinely stressors [12–14], can increase activities of the masticatory muscles and be associated with TMD[14]. Surely, some functional disorders affecting the stomatognathic system have promoted painful sensibility and discomfort in masticatory muscles [10]. This muscle adaptation has been analyzed after unilateral extraction [15–18][15, 16, 17, 19] and other occlusal interferences.

Oxidative stress may be part of morphological changes in masticatory muscles due to the fact that these areas are submitted to multiple trauma and inflammatory processes. The synthesis of antioxidants has shown to prevent muscle degeneration [18]. Thus, the production of reactive oxygen species (ROS) can be changed during orofacial muscle dysfunction [19]. In addition, ROS are known to participate in multiple signaling pathways that are important for homeostasis and the adaptation of skeletal muscle cells [20].

Reactions with nicotinamide adenine dinucleotide diaphorase (NADH) and succinate dehydrogenase (SDH) were used to demonstrate the oxidative capacity and metabolic activity of muscles. In these reactions, nitroblue tetrazolium is exposed to the substrate, acting as a major acceptor of hydrogen [21]. Through the intensity of the staining of a muscle, as well as light, intermediate and dark fibers, it is possible to analyze the modified plasticity in a masticatory muscle.

To better understand the pathophysiology mechanisms of masseter muscle dysfunction in the presence of possible causal factors of TMD pain, the present study assessed the effects of acute stress and unilateral edentulism on the metabolism and oxidative stress of this muscle. The two factors of variation are the conditions of high incidence and prevalence in the general population. Nevertheless, this study is pioneering in accessing the influence of the two factors together under the morphophysiology of a masticatory muscle.

## Materials and Methods

### Animals

Experiments were performed with Wistar male rats weighting 275 ± 300g and were obtained from the University of São Paulo, Campus of Ribeirão Preto, Brazil. The animals were housed in a temperature-controlled room (24 ± 1°C) and 12-h light/dark cycles (lights on at 06:00 am) with food and water ad libitum. All experimental procedures were approved by the Animal Use and Ethics Committee of Ribeirão Preto, University of Sao Paulo (Process number 11.1.130.53.5). All efforts were made to minimize animal suffering and to reduce the number of animals used.

The animals were randomly divided into 4 groups: a group with extraction and acute stress (E+A); group with extraction and without stress (E+C); group without extraction and with acute stress (NO+A); and control group without both extraction and stress (NO+C).

### Induction of Modified Occlusion by unilateral Edentulism

On day zero, the rats were subjected to the extraction of the upper left molars. The animals were anesthetized with an association of 4% Xylazine (10 mg/kg) and Ketamine (80 mg/kg). The extractions were performed by using an anatomical clamp and the Hollemback 3S (child), disinfected with iodine solution. As a prophylactic measure, the animals received a single dose of an antibiotic (Pentabiotic veterinary—“Fort Dodge”) in a dose of 24 mm IU of penicillin per kg of body weight, as well as anti-inflammatory and analgesic Bananine (Schering-Plorigh, flumexine meglumine, 25 mg/kg, 10 mg/ml). Considering the surgical stress induced by the extraction, in the NO groups, a simulation of the extraction process was conducted, so the rats were anesthetized and received the same dose of antibiotic and anti-inflammatory treatments.

### Acute Stress Protocol

This protocol consisted of a single episode of stress performed on day 23^rd^. Ten animals were submitted to the protocol of acute stress on day 23^rd^ after the zero time, with or without molar extraction. The animals were placed in a metal box, which was 15 cm long and 5 cm in diameter and had adequate ventilation throughout its length. The end of the box was closed, and the animals were in a state of physical restraint for 2 hours, which restricted their movements.

### Obtaining Samples

On day 23^rd^, all rats were previously anesthetized with 4% Xylazine (10 mg/kg) and 10% Ketamine (80 mg/kg) and then submitted to euthanasia. On rats undertaking the acute stress, the euthanasia were performed after the stress. The animal’s fragment of the middle third of the deep masseter muscle was dissected and frozen in liquid nitrogen (-150°C). Samples were stored in a freezer at -80°C and were then transferred to a cryostat microtome chamber at -20°C to conduct the cutting of sections. The sections taken for all analysis of the present work were removed of the left side of experimental animals.

### Histochemical Technique for Demonstration of Succinate Dehydrogenase (SDH)

To evaluate metabolic activity, a histochemical technique was performed for the demonstration of succinate dehydrogenase (SDH) activity. To reveal the fiber type, cross-sections of 10 μm thickeness were subjected to a reaction in a solution containing 0.2 M sodium succinate as a substrate and nitroblue tetrazolium. With the aid of a camera attached to an OLYMPUS BX 61 microscope, 20 images from each group were systemically captured with a 20x lens. A system test of 80 points was superimposed on each image to quantify the points for each fiber type—high, intermediate and low metabolic activity—by using Image J software (National Institutes of Health). Relative areas occupied by fiber types, with consideration of the dark fibers as the most oxidative metabolic activity, determined the metabolic activity of this muscle.

### Histochemical Technique for Demonstration of Nicotinamide Adenine Dinucleotide Diaphorase (NADH)

Slides of cut muscled were incubated in a solution containing NADH at 37°C for 30 minutes. Subsequently, they were washed with distilled water, fixed in 5% formaldehyde that was buffered at pH 7.0 for 5 minutes, washed again with distilled water and mounted with Entellan. From each cross section of a muscle, 5 images were systematically captured with a 20x lens. A system test of 80 points was superimposed on each image to quantify the points for each fiber type—dark, intermediate and clear—by using Image J software (National Institutes of Health).

### Histochemical Technique for Reactive Oxygen Species (ROS)

Slides containing cross-sections of 10 μm thick were incubated with DHE for 30 minutes in a humid and dark chamber. After incubation, the sections were washed with phosphate-buffered saline (PBS) and fixed in 4% PFA. The dihidroetideo (DHE) was used at a concentration of 10 mg/mL [22, 23]. This "probe" will react with superoxide (O_2_) that is present in the tissues, which results in the formation of 2-hidroxietideo and ethidium, products that emit red fluorescence on the spot. The parameter evaluated was the quantification of the concentrations of reactive oxygen species produced by the muscles in each experimental protocol. By using a fluorescence microscope (Leica Imaging Systems Ltd., Cambridge, UK) coupled to a camera; the images of the slices were photographed at 500x. The quantification of the intensity of emitted red fluorescence was performed by using Image J software (National Institutes of Health).

### Statistical Analysis

To observe data normality, groups were compared by two-way ANOVA with fixed factors of variation (acute stress and exodontia), followed by a Tukey-Kramer *post-hoc* test. The P < 0.05 was considered statically significant.

## Results

### Succinate Dehydrogenase (SDH)

**Fig 1:**
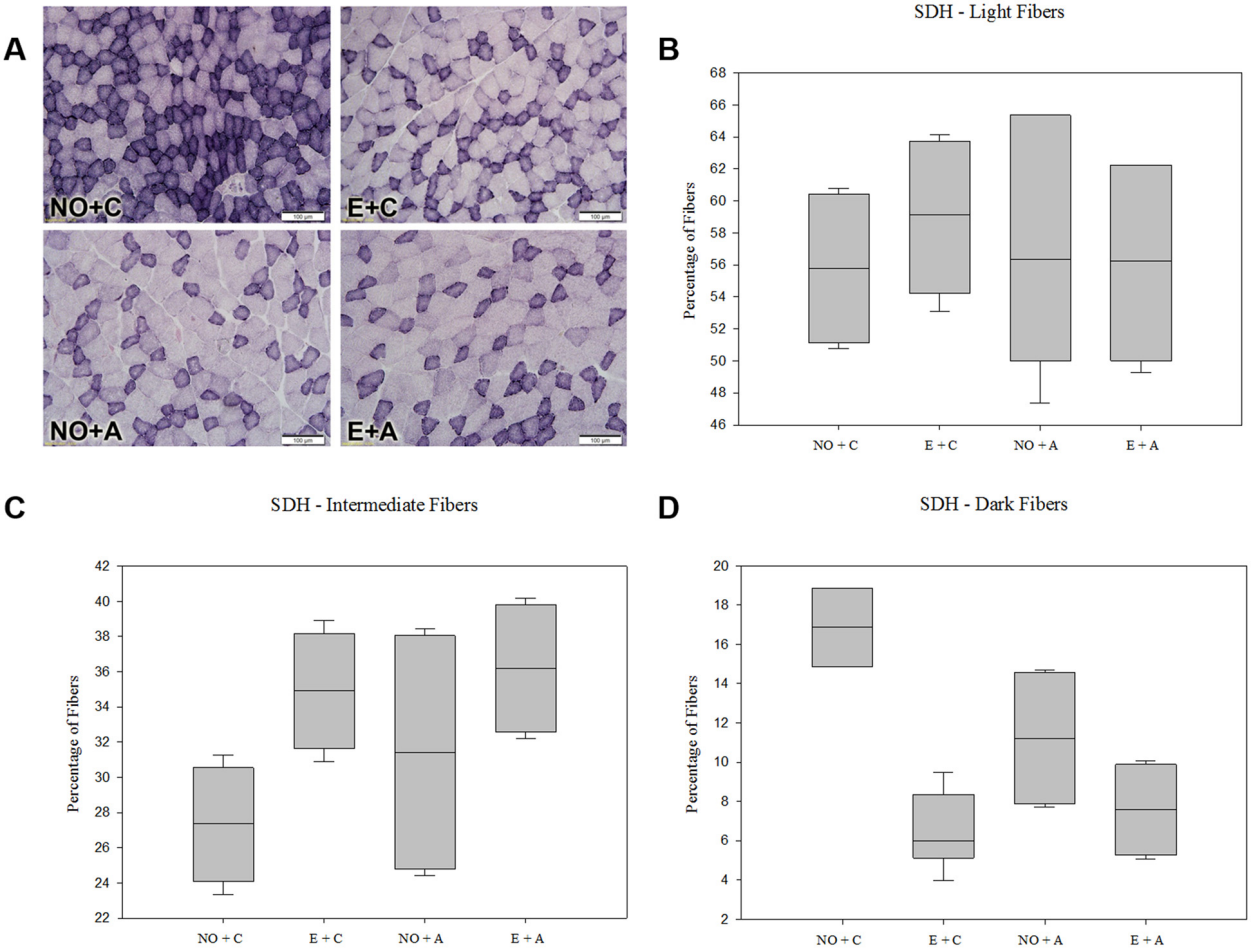
Demonstration of succinate dehydrogenase (SDH) in different groups of rats. Panel A, photomicrographs of the masseter muscle of rats. 200x magnification. Magnification bars: 100 μm. Panels B, C and D means the percentage of Light Fibers (B), Intermediate Fibers (C) and Dark Fibers (D) in different groups. Fig 1D: P < 0.05, NO+C group *vs* E+C, NO+A and E+A groups (Tukey-Kramer test).

The reaction of the SDH with nitro-bluetetrazole (Nitro-BT) showed the masseter muscle constituted by fibers with high, intermediate and low dehydrogenase activities (Fig 1A, 1B, 1C and 1D). These activities were evaluated according to the staining intensity of the fibers and because of the pigments deposited in the mitochondria (Fig 1A). The percentage of relative areas of deeply stained fiber decreased in the group submitted to unilateral extraction (E), (5.98% ± 1.62, P < 0.05), acute stress (A) (11.21% ± 2.01, P < 0.05) or the association of both factors (E+A) (7.57% ± 1.98, P < 0.05), compared to the normal occlusion (NO+C) group (16.87% ± 2.04) (Fig 1D). Meanwhile, the areas of weakly stained fibers showed to be unaffected under the same conditions of the experiment. However, the relative areas of intermediately stained fibers showed a tendency to increase. These data indicate that both exodontia and acute stress, whether isolated or associated, affected succinate dehydrogenase activity in the muscle.

### Nicotinamide Adenine Dinucleotide Diaphorase (NADH)

**Fig 2:**
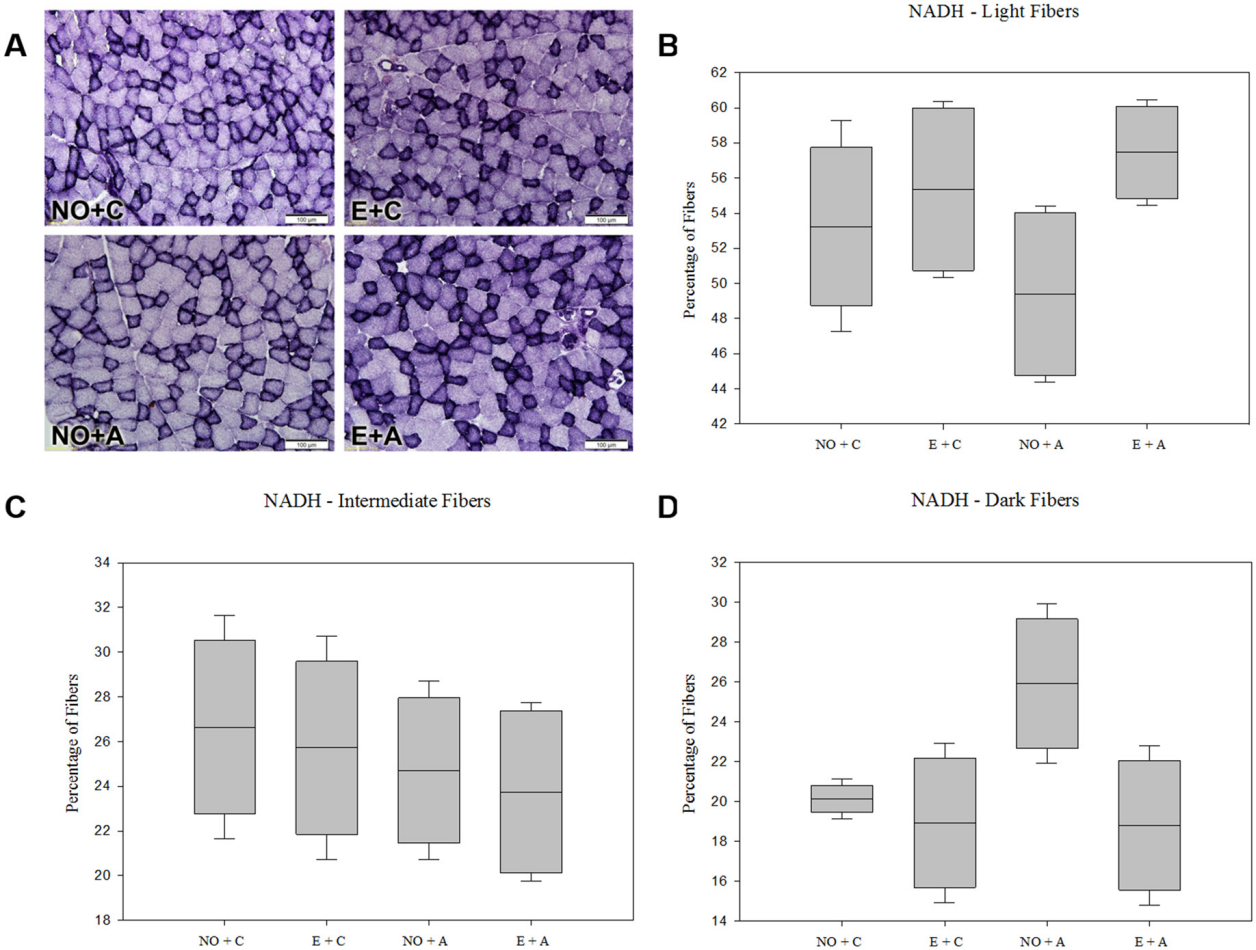
Demonstration of Nicotinamide Adenine Dinucleotide Diaphorase (NADH) in different groups of rats. Panel A, photomicrographs of the masseter muscle of rats. 200x magnification. Magnification bars: 100 μm. Panels B, C and D means the percentage of Light Fibers (B), Intermediate Fibers (C) and Dark Fibers (D) in different groups.

The masseter muscle showed oxidative fibers with products that were fine-grained and scattered throughout the cytoplasma (Fig 2A). The oxidative-glycolytic fibers had thick subsarcolemmal granules and glycolytic fibers were less stained. Regarding the NADH diaforase reaction, the percentage of relative areas of dark, intermediate and light fibers did not change significantly in the rats submitted (E+A; NO+A) or not to acute stress (E+C; NO+C) (P > 0.05, Fig 2B, 2C and 2D). Neither the modified occlusion nor the acute stress altered the oxidative activity of the masseter muscle’s posterior fibers.

### Reactive Oxygen Species (ROS)

**Fig 3:**
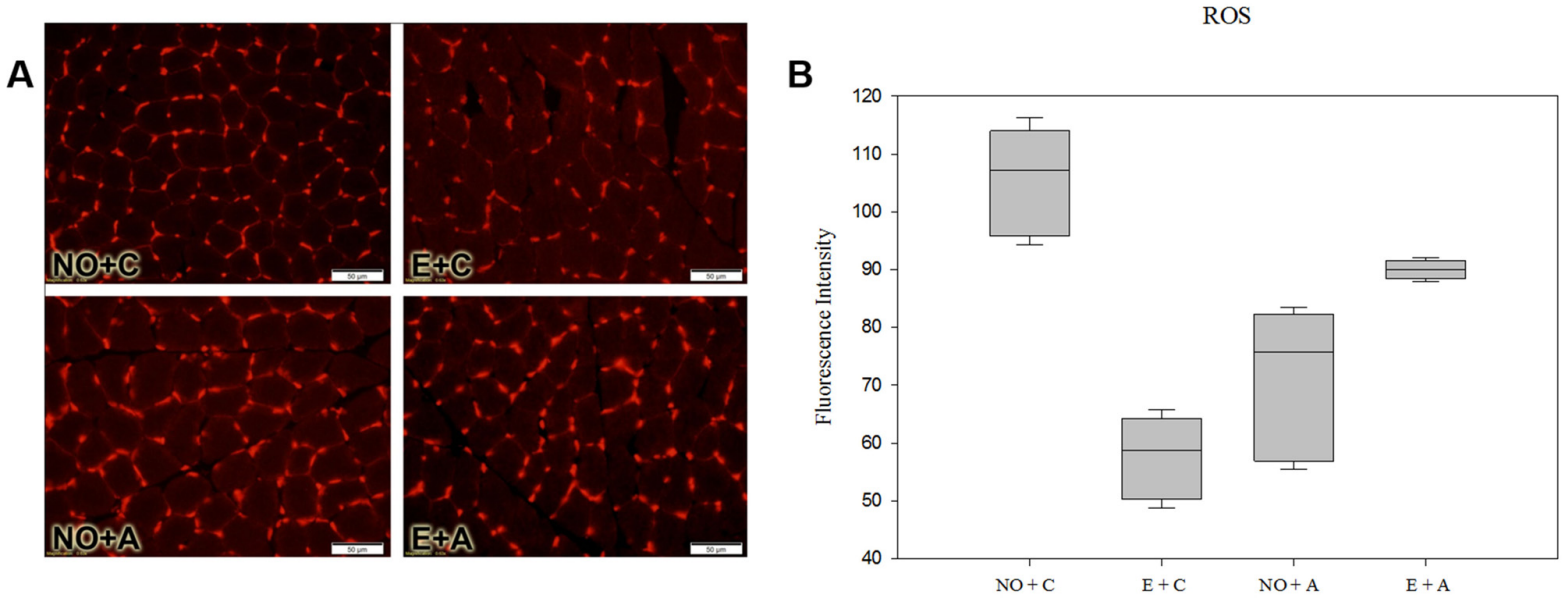
Quantification of Reactive Oxygen Species (ROS) in different group of rats. Panel A, photomicrographs with fluorescent labeled for ROS of masseter muscle of rats. 200x magnification. Magnification bars: 50 μm. Panels B, C and D means de fluorescence intensity of ROS in differente groups. Fig 3B: P < 0.05, NO+C group *vs* E+C, NO+A and E+A groups; and P < 0.05, E+A group *vs* E+C group (Tukey-Kramer test).

The fluorescence intensity revealed the reactive oxygen species (ROS) in the different groups (Fig 3A). The two-way ANOVA indicated that in the E+C group (57.75 ± 3.27), NO+A group (71.6 ± 6.1) and E+A group (90 ± 0.70), there was a decrease in the expression of ROS (P < 0.001, Tukey-Kramer, Fig 3B), compared to the NO+C group (105.25 ± 4.9 arbitrary units).

All results of SDH, NADH and ROS with respective delta scores were summarized in the following table (Table 1).

**Table 1:** Percentage of the three different fiber types identified by SDH and NADH reactions and average of ROS in the left masseter muscle in rats.

Histochemical Technique	Fiber Types	Experimental Groups	Average	Percentage	SEM
**SDH**	Light Fibers	NO + C		55.78	2.13
		E + C		59.12	2.53
		NO + A		57.37	4.29
		E + A		56.25	3.45
	Intermediate Fibers	NO + C		27.35	1.82
		E + C		34.9	1.52
		NO + A		31.42	3.99
		E + A		36.18	1.52
	Dark Fibers	NO + C		16.87	2.04
		E + C		5.98*	1.62
		NO + A		11.21*	2.01
		E + A		7.57*	1.98
**NADH**	Light Fibers	NO + C		53.24	2.03
		E + C		55.36	1.96
		NO + A		49.38	1.55
		E + A		57.46	1.1
	Intermediate Fibers	NO + C		26.64	2.75
		E + C		25.72	1.17
		NO + A		24.71	1.91
		E + A		23.74	1.95
	Dark Fibers	NO + C		20.12	0.46
		E + C		18.92	1.39
		NO + A		25.91	1.69
		E + A		18.8	1.59
**ROS**		NO + C	105.25		4.9
		E + C	57.75* ^a^		3.27
		NO + A	71.6*		6.1
		E + A	90.0*		0.7

NO + C: control group without both extraction and stress; E + C: group with extraction and without stress; NO + A: group without extraction and with acute stress and E + A: group with extraction and acute stress. SEM: Standart Error Means. The Two-way Anova statistical analysis reveals that in SDH technique, the percentage of relative areas of dark fibers decrease in the group submitted to unilateral extraction, acute stress or the association of both factors compared to control group, whereas NADH results didn’t show statistical differences between the groups. Furthermore, the ROS expression showed a decrease in all experimental groups analyzed compared to the NO + C group.

* P<0.05 Tukey-Kramer post-hoc test when compared with control group.

^a^ P<0.05 Tukey-Kramer post-hoc test when compared E + C with E + A with group. Delta scores for each percentage of fibers (SDH and NADH) or average of fluorescence intensity (ROS) is indicated in the table.

## Discussion

Occlusal interferences are associated with short-term clinical signs and symptoms, such as masticatory muscle fatigue and pain [24]. Many dentistry researchers and practitioners believe that muscle dysfunction plays a significant role in the origin of TMD. Masseter muscle is considered to be one of the most important muscles of mastication [25] and it can be affected by idiopathic or pathophysiological conditions with or without the presence of pain [26]. An imbalance of the harmonious interaction between maxillary and mandibular dental elements can lead to the collapse of structures in the stomatognathic system. Moreover, an adaptation of muscles and adjacent structures can occur without compromising the normal functions of this system [27, 28]. However, it has long been believed that modified occlusion may affect not only the morphology but also the masticatory functions [29, 30] of the jaws and face. With an aim to promote an understanding of the pathophysiology of muscle disorders, this study evaluated the effects of a physical stressful situation previously used by some researchers [31], as well as unilateral edentulism on metabolism and oxidative stress in masseter muscle.

The SDH enzyme in the histological section releases hydrogen from the succinate and it is an important marker of metabolic activity. The mitochondrial SDH complex catalyzes the oxidation of succinate to fumarate substrate in the Krebs cycle and supplies the ubiquinone population of the respiratory chain [32]. Therefore, the mitochondrial SDH is a functional member of the electron transport chain [33], which plays a central role and is indispensable in several tissues and cells, such as muscles cells [34]. In the present study, the reduction of relative areas of dark fibers was evident in the medial masseter muscles of the groups that were submitted to unilateral extraction, acute stress or an association of both factors. However, the group that was subjected to extraction showed smaller relative areas of these dark fibers, when compared with groups treated with other factors; this indicate that occlusal alteration by teeth extraction decreases the vertical dimension of occlusion and contributes to muscle dysfunction. Corroborating these findings, Bazan et al. [16] reported that tooth extraction decreased the metabolic activity of an ipsilateral medial pterygoid muscle of a guinea pig; Iyomasa et al. [35] showed that after 60 days of unilateral extraction there are muscle fibers of small diameter and central nuclei in the medial pterygoid muscle of gerbil. The authors also showed that the reduction in the vertical dimension of occlusion by a loss of posterior teeth changes the position of fibers in the masseter and medial pterygoid [36].

Stress by restriction is a well-characterized model that was used in this study to induce acute stress, which decreased the activity of SDH. Interestingly, although previous studies verify the influence of occlusal alterations in SDH activities in masseter muscle, this is the first investigation that relation metabolic activity by SDH in this muscle with a condition of stress. The reduction of dark fibers indicated in this work may be due to the increased activity of the hypothalamic pituitary adrenal (HPA) axis by stress, which in turn increases the levels of glucocorticoids [37]. Glucocorticoids are important and have complex effects on glucose metabolism, causing hyperglycemia and decreased glucose uptake by tissues, including muscles [38]. It is worth noting that a glucocorticoid’s response to acute stress is important for the whole body, homeostasis and the survival of the species. However, this situation can lead to chronic pathophysiological states. Naturally, it is possible that a decrease in long SDH can be a pathophysiological factor for TMD or masticatory muscle dysfunctions.

This investigation used a histochemical reaction for NADH for the analysis of muscle metabolism too. It is possible to investigate metabolic capacity for ATP synthesis, according to three types of fiber staining: dark (oxidative glycolytic) with a high capacity to aerobic and anaerobic metabolism; intermediate (oxidative), which depends on aerobic metabolism; and light (glycolytic), which metabolism occurs in situations with low levels of oxygen [39].

Observations of masseter muscle section in this work reveal that the exodontia, stress alone or stress associated with the teeth extraction did not alter the metabolic capacity of muscle fibers significantly. However, it was noted that in the group subjected to stress without extraction, there was a tendency to increase the relative areas of glycolytic fibers, due to the tendency to reduce the areas of dark fibers. These data could suggest that there was decreased oxidative metabolic activity of the muscle region analyzed. Miehe et al. [40] who performed a bilateral extraction of teeth associated with a soft diet in rats, which increases the number of glycolytic fibers, indicating smaller force chewing and decreased oxidative capacity, can support this suggestion. According to White and Schenk [41], during muscle contraction, levels of NADH decrease. In addition, stress and occlusal interference influence the electromyographic activity of some chewing muscles during a mastication cycle [42]. However, there are no studies indicating the effects of altered occlusion and a stressful situation on metabolic activity by NADH in masseter muscle, indicating that this is the first study to examine this interaction and that there is need for further studies to strengthen this suggestion for NADH results.

The detection of ROS production by skeletal muscle cells is fundamental to the problem of differentiating between physiological and pathological levels [43]. In the present study, the altered occlusion that were either associated or not associated with acute stress, as well as acute stress alone, showed a decrease in ROS expression. Our data corroborate Li et al. [44], that found that stress induced by electric shocks on a rat’s paws results in oxidative damage and changes in the regulation of antioxidant proteins in the masseter muscle of a rat. In addition, about to masseter muscle of patients with painful symptoms by TMD, it was observed reduced levels of oxidative stress in samples of this muscle compared with patients free of pain. Moreover, oxidative stress has been associated with intensity of joint and muscle pain, suggesting that oxidative stress contributes to pain in symptomatic TMD patients [45].

Although the mitochondria is commonly the predominant source of ROS in skeletal muscle cells, Piao et al. [46] also considered other sources-such as NADPH oxidases that are located within the sarcoplasmic reticulum, transverse tubules and sarcolemma. Indeed, ROS may be associated with the induction of muscle atrophy in autophagy [47]. Nonetheless, not all studies have concluded that ROS production in an inactive skeletal muscle plays an important role in disuse muscle atrophy [48].

Despite of observation of significant ROS reduction in all treated groups of this work compared to control group, the results for E+A needs attention, since the factor stress in this group seems to revert the effect of exodontia. When comparing only the effect of exodontia with a combination of the effects of these two factors, it is suggested that acute stress for two hours caused greater impact on oxidative stress than in muscle metabolic activities by SDH and NADH. So, masseter muscle seems to be more fragile and therefore with greater production of reactive oxygen species. In the same way, Cutando et al. [49], showed an increase in oxidative stress, verified through ROS production in plasma, after 24 hours of maxillary and mandibular premolars and molar extraction. This short time analysis can suggest not only the factor of tooth removal, but also, the stress effect of post-surgery time, resulting in an influence of the two factors association in an increase of ROS. Our work opens the way for the study of role of ROS in masticatory muscle dysfunction. Therefore, this theme remains little explored, and other studies should be conducted to better understand its pathogenesis.

Thus, this study is pioneer in assessing acute stress and exodontia together as factors that influence the oxidative metabolism of the masseter muscle of rats. Accordingly, the metabolic activity of muscle fibers was reduced by those factors. Nevertheless, the extraction was a predominant etiological factor that affected the metabolic plasticity of the masseter muscle at the molecular level, possibly contributing to inactivity-induced masticatory muscle atrophy. Despite the decrease of ROS in the muscles submitted to both factors in this study, there is growing evidence suggesting that muscle inactivity- which led to reduced production of ROS- played an important role in the rate of disuse skeletal muscle atrophy. Studies on the signaling pathway that connects ROS and dysfunctions of masticatory muscles—involving different ways of stresses applied to an experimental model—remain an important topic for further research. These studies can lead to the identification of biological targets for therapeutic intervention to protect against muscle and temporomandibular dysfunction.
